# Catheter Ablation in Complex Atrial Arrhythmias: Pilot Study Evaluating a 3D Wide-Band Dielectric Imaging System

**DOI:** 10.3389/fcvm.2021.817299

**Published:** 2022-01-28

**Authors:** Janis Pongratz, Uwe Dorwarth, Lukas Riess, Yitzhack Schwartz, Michael Wankerl, Ellen Hoffmann, Florian Straube

**Affiliations:** ^1^Department of Cardiology and Internal Intensive Care Medicine, Heart Center Munich-Bogenhausen, Munich Clinic Bogenhausen, Academic Teaching Hospital of the Technical University Munich, Munich, Germany; ^2^EPD Research, Caesarea, Israel; ^3^Medical Faculty, Ludwig-Maximilians-University, Munich, Germany

**Keywords:** dielectricity, atrial tachycardia, atrial fibrillation, cryoballoon ablation, dielectric, atrial flutter, electro-anatomical mapping

## Abstract

**Background:**

Cryoballoon ablation (CBA) for pulmonary vein isolation (PVI) is a standard in atrial fibrillation (AF) ablation but might not be enough in complex atrial arrhythmias (AA). An open three-dimensional wide-band dielectric imaging system (3D-WBDIS) has been introduced to guide CBA.

**Material and Methods:**

Pilot study evaluating feasibility and safety of 3D-WBDIS in combination with CBA and optional radiofrequency ablation (RFA) in patients with complex AA defined as (1) history of persistent AF, (2) additional atrial tachycardia/flutter, or (3) previous left atrial ablation.

**Results:**

Prospectively, seventeen patients, 68.9 ± 12.2 years of age, with complex AA were enrolled. In 70 pulmonary veins (PV), balloon positioning maneuvers (*n* = 129) were guided additionally by the occlusion tool (1.84/PV). Compared to angiography, its sensitivity and specificity was 94.5, and 85%, respectively. CBA-PVI was achieved in 100% of PVs including variants. In 68 maps, the median number of mapping points was 251.0 (interquartile range (IQR) 298.0) with a median map volume of 52.8 (IQR 83.9) mL. Following CBA, six additional arrhythmias (two right and two left atrial flutter, one left atrial appendage tachycardia, and one atrioventricular nodal reentry tachycardia) were identified and successfully ablated by means of RFA in five patients (29.4%). Left atrial and fluoroscopy times were 88 (IQR 40) and 20 (IQR 10) minutes, respectively. Dose area product was 1,100 (IQR 1252) cGyxcm^2^. Freedom from AA after 6 months follow-up time and 90 days blanking period was documented in 10/17 (59%) patients, and 8/17 (47%) without a blanking period. No major complication was observed.

**Conclusion:**

The combined use of CBA with optional RFA guided by a novel 3D-WBDIS is feasible and safe in patients suffering from complex AA. The occlusion tool shows high sensitivity and specificity for assessment of the balloon occlusion. Additional arrhythmias were successfully mapped and ablated. Short-term outcome is promising, and subsequent prospective, larger outcome studies are necessary to confirm our observations.

## Introduction

The interventional treatment of atrial fibrillation (AF) has a strong level of recommendation in current guidelines as it shows superiority compared to anti-arrhythmic drug treatment, improves symptoms, and quality of life (1, 2). Moreover, catheter ablation in heart failure patients with AF can improve prognosis (3), and early rhythm-control therapy was associated with a lower risk of adverse cardiovascular outcomes than usual care (4). AF is initiated mainly by ectopic beats originated from the pulmonary veins (PV). Thus, pulmonary vein isolation (PVI) by means of radiofrequency ablation (RFA) or cryoballoon ablation (CBA) is recommended with a class I indication. In June 2021, the Arctic Front Advance™ CB system (Medtronic Inc., MN, United States) has received expanded FDA approval for first-line treatment of paroxysmal AF (PAF) without prior antiarrhythmic drug treatment based on the results of the Cryo-FIRST (5), the STOP-AF (6), and the EARLY-AF (7) results. The use of additional ablation lesions beyond PVI (low voltage areas, lines, fragmented activity, ectopic foci, rotors, and others) may be considered but is not well-established (class IIb recommendation) (1, 2).

CBA provides safe and effective PVI and the durability of the PVI is high in paroxysmal and persistent AF (8–11). In patients with structural heart disease, an underlying atrial substrate often exists and its extent predicts the long-term success of AF ablation (12). In patients with atrial arrhythmias (AA) despite durably isolated veins after CBA, atrial tachycardia (AT) or atrial flutter are frequently observed and treated in a second left atrial (LA) ablation procedure (8). In clinical practice, patients suffering from more complex forms of AA might be identified clinically due to the history of arrhythmias, the documented electrocardiogram (ECG), well-known clinical predictors and the use of pre-procedural imaging (e.g., LA size) (13, 14).

A novel open platform three-dimensional Wide-Band Dielectric Imaging System (3D-WBDIS, KODEX EPD, EPD solutions, a PHILIPS company, The Netherlands) based on the evaluation of dielectric information of the catheters, and the tissue has been introduced (15, 16). Besides the standard tissue contact-based activation and voltage mapping, the visualization of the anatomic shell does not need direct catheter-tissue contact, and any catheter can be used and visualized in combination with the system (15). Moreover, the occlusion of the PV with the cryoballoon (CB) catheter immediately before the initiation of a freeze cycle can be guided and determined with the system by measuring induced change in dielectric properties during positioning of the balloon over the spiral mapping catheter (SMC; Achieve Advance™, Medtronic Inc., Minneapolis, MN, United States) (17, 18). The system has the potential to reduce radiation exposure of the patient and the lab staff. No data exist on the feasibility of the system to map and treat additional arrhythmias by means of RFA.

CBA became widely accepted especially due to the straightforward procedure. In experienced hands the first PV is isolated in 3 min after the transseptal puncture by means of standard CBA (without 3D-WBDIS). This fast and efficacious strategy with high rates of durable PVI has also emerged as a strategy in persistent AF. In a conventional setting without the use of the 3D-WBDIS radiation dose and LA time were brought down to values of 800 cGyxcm^2^ and 50 min, respectively (10).

In the present study, and in a special subset of patients which might require more than a sole PVI, our aim was to perform (1) a very straight forward PVI procedure using CB-technology with the latest occlusion tool technique under guidance of the 3D-WBDIS, and (2) to identify and treat additional arrhythmias after the completion of the CBA by means RFA with the benefit of a 3D electro-anatomical mapping (EAM) technology.

The main purpose of the pilot study was to evaluate the technical feasibility of the 3D guidance of CBA and RFA in patients suffering from complex AA which might require additional ablation beyond sole PVI. Procedural results, complications, and short-term outcome results of this strategy were evaluated in this study.

## Methods

Consecutive symptomatic AF patients with complex AA scheduled for the first AF ablation procedure were prospectively included into the observational single-center registry study.

“Complex AA” was defined as (1) history of persistent AF (duration of a single episode of 7 days up to 12 months), (2) additional AT/atrial flutter, or (3) previous LA ablation. Exclusion criteria were according to the guidelines (19). Patients with LA diameter >60 mm were not referred for AF ablation. Prior to the procedure, baseline characteristics were documented, and all the patients underwent transthoracic and transesophageal echocardiography. Hypertension was defined as blood pressure values higher than 140/90 mmHg following the European Society of Cardiology (ESC) and the European Society of Hypertension (ESH) guideline for the management of arterial hypertension (20). Hypertensive heart disease was defined as left ventricular hypertrophy (>12 mm intraventricular septum) and diastolic dysfunction determined by transthoracic echocardiography. “Prior myocardial infarction” was documented in those patients with previously diagnosed coronary artery disease and a history of myocardial infarction defined as ST segment elevation myocardial infarction or non-ST segment elevation myocardial infarction before enrollment. Last intake of direct acting anticoagulants was in the evening before the procedure and restarted 30–60 min after sheath removal. Those patients on vitamin-K antagonists were ablated on treatment with targeted International Normalized Ratio between 2.0 and 2.5.

The primary strategy of ablation was to isolate all PV by means of CBA with additional use of the 3D-WBDIS (KODEX EPD, EPD solutions, a PHILIPS company, The Netherlands) and evaluation of the provided occlusion tool. Electro-cardioversion was performed in case of intraprocedural AF after transseptal puncture and before the balloon catheter was turned to the septal PVs as phrenic nerve stimulation while ablating the right sided PVs requires repositioning of the decapolar diagnostic catheter from the coronary sinus (CS) to the superior vena cava. After acute PVI, a left atrial 3D EAM including the anatomic shell, an activation, and voltage map was acquired using the SMC in sinus rhythm, paced or during tachycardia if present. Right atrial maps were added if deemed necessary. Procedural data, periprocedural results including complications were documented, and short-term follow-up data are provided.

### Description of the Novel 3D-WBDIS

The 3D-WBDIS uses a set of three pairs of body surface sensors and a right-leg reference sensor, to induce an intra-body global electrical field. The system continuously collects the electromagnetic signals at 100 Hz sampling rate from all patches and electrodes. This information is used for tracking and navigation of any standard diagnostic or therapeutic EP catheter not equipped with a magnetic location sensor, as well as for imaging and acquiring an electro-anatomical map of the cardiac chamber. Furthermore, a second induced set of intra-body local electrical fields, whereby each electrode emits and receives an electrical field, is used for interrogation and characterization of the local field shape and tissue signatures (dielectric mapping). For example, there are 16 different measured voltages and 16 corresponding calculated impedances for a typical 4-electrode catheter. These are used to generate the fine cardiac chamber images and the tissue signatures.

The 3D-WBDIS differentiates materials based on their dielectric properties. The dielectric properties of a material are determined by its conductivity and permittivity, relative to the frequency of the electrical field. Conductivity is a measure of a material's ability to conduct electrical currents. Permittivity quantifies its ability to polarize within an electrical field. By measuring the conductivity and permittivity at multiple frequencies (10–100 kiloherz), it is possible to establish a dielectric dispersion pattern. In biological tissues the dielectric dispersion pattern is determined by its constituents at a cellular and molecular level. In cardiomyocytes for example, the intracellular fluid is an important conductor, while the sarcoplasmic reticulum contributes to the permittivity (21).

Structures such as the endocardial atrial surface, cardiac veins, and heart valves cause marked gradients in the electrical field (16). This “bending of the electrical field” is sensed by the system and used to calculate the geometric characteristics of the 3D image. The 3D-WBDIS rapidly generates high-resolution 3D images of cardiac anatomy obviating the need for direct physical contact between the catheter and the endocardial surface or pre-acquired magnetic resonance or computed tomography images (16).

Intracardiac electrograms can be recorded from the catheter to create local activation time (LAT) and propagation maps, bipolar voltages can be recorded to show voltage maps as most widely used with other systems. Ablation parameter visualization and tagging tools are available as the system is connected to the used RF generator (Smartablate®, Biosense Webster Inc., California, United States). The 3D-WBDIS provides catheter stability, intracardiac electrical activation information, and during ablation, impedance drop, and temperature as read from the RF generator. Ablations point tagging is conducted based on a user defined combination of parameters and thresholds. In addition, the system provides power, power integral over ablation time and duration.

### Cryoballoon Procedure

The procedure was performed in deep analogosedation. After venous access, a decapolar steerable mapping catheter (Biosense Webster Inc., California, United States) was introduced into the CS. A single transseptal puncture was performed over a guiding sheath with a BRK™ XS needle (Abbott, Illinois, United States) and was guided by intracardiac echocardiography (ICE; ACUSON AcuNav™, Biosense Webster Inc., California, United States). The ICE catheter was placed in the right atrium throughout the procedure to visualize the interatrial septum, the transseptal puncture, the sheath with the catheters in 2D mode or if necessary, with color Doppler imaging.

The steerable sheath (Flexcath Advance™, Medtronic Inc., Minneapolis, MN, United States) was positioned in LA over a stiff guidewire. The CB (Arctic Front Advance Pro™, Medtronic Inc., Minneapolis, MN, United States) was introduced over the SMC with a fixed diameter of 20 mm and 8 electrodes and positioned at the ostium of the PV. Activated clotting time was kept between 300 and 400 s with unfractionated heparin. PV potentials must have been recorded at least before and after complete PVI with a circular mapping catheter. Periprocedural management was performed in accordance with the current practice guidelines (19). Balloon positioning was visualized with fluoroscopy, ICE, and the 3D-WBDIS. Immediately before the start of the cryoablation cycle, the occlusion was determined with contrast injection through the inner lumen of the CB.

An endoluminal esophageal sinusoidal temperature probe with 12 thermocouples (CIRCA S-Cath™, CIRCA Scientific, Englewood, CO, United States) was used to monitor temperatures. Phrenic nerve (PN) stimulation was controlled by palpation and ICE visualization of the diaphragmatic motion. For premature termination due to esophageal temperatures ≤ +15°C, PN impairment or balloon temperatures ≤ -55°C, double stop technique was applied.

A time-to-isolation (TTI) guided cryodosing protocol was used in all cases. Standard freezing time was set at 180 s. An additional application was performed after acute electrical disconnection if a TTI ≧45 s was observed or if no TTI was documented.

### The Use of the Occlusion Tool

**Figure 1:**
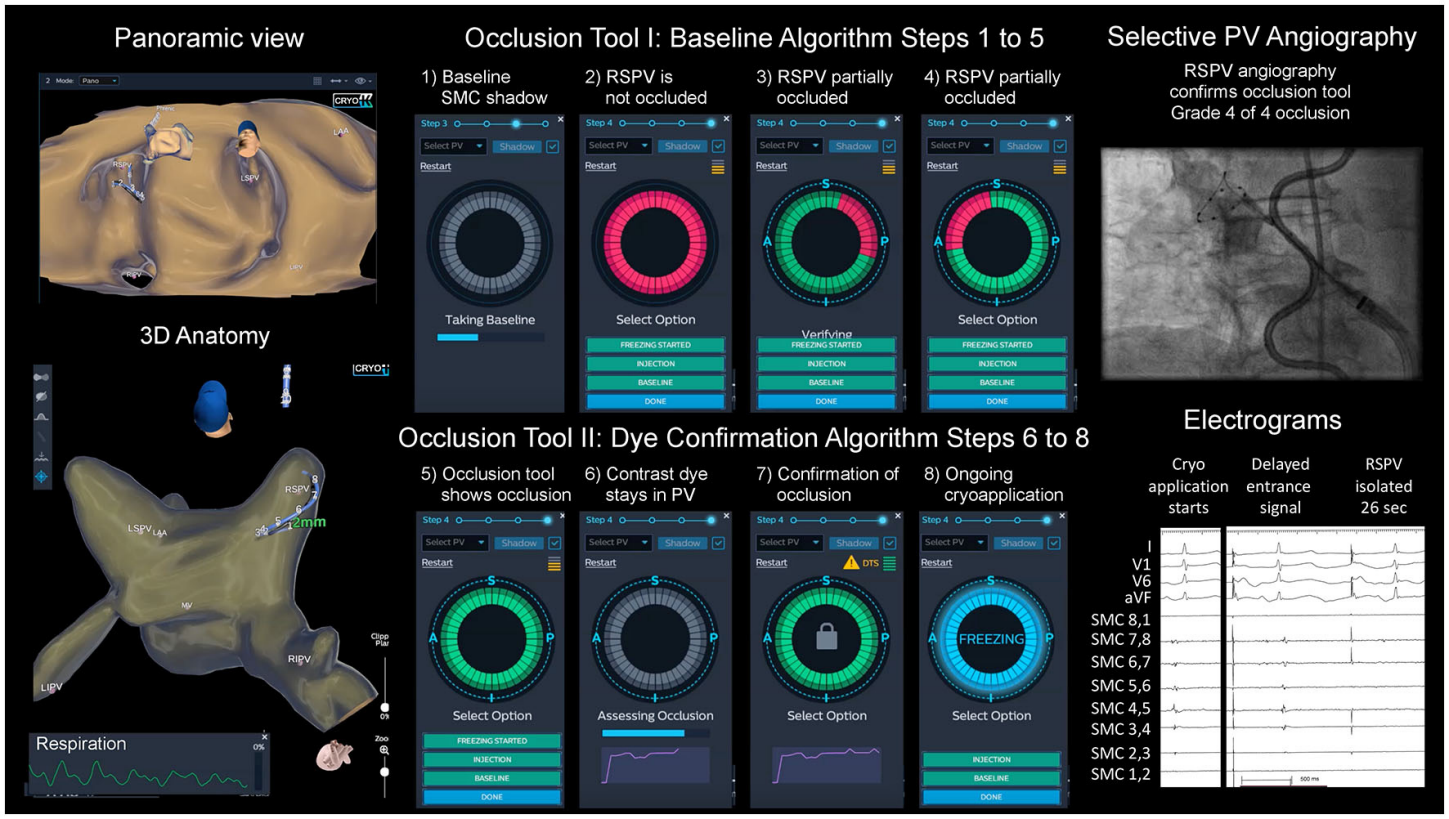
Cryoballoon ablation guided by dielectrical information of the occlusion tool. Left atrial dielectrical anatomy map (left upper hand: panoramic view, left lower hand: posterior view) showing typical pulmonary vein (PV) anatomy. Step-by-step workflow of balloon positioning guided by the occlusion tool (1–8). Right upper hand: angiography of right superior PV (RSPV) with spiral mapping catheter (SMC) placed in RSPV, endoluminal esophageal sinusoidal temperature probe, multipolar catheter in superior vena cava for phrenic nerve pacing; right lower hand: acute PV isolation electrograms with disappearance of the entrance signal after 26 s, artifacts from phrenic nerve pacing.

The occlusion tool was used to guide PVI as a supplement to standard angiography. Over the SMC, the inflated CB was positioned at the ostium of the PV without occluding the PV. The SMC was pulled back close to the proximal PV position and a “shadow” was acquired to mark the baseline position. The SMC needs to be kept within </=10 mm from its “shadow” throughout the occlusion testing. Afterwards, the balloon was advanced to the PV ostium, aiming at complete occlusion. The system, measuring the change in local dielectrics induced by the presence of the large non-conducting balloon, provided qualitative color-coded assessment of the occlusion state. A full green circle symbolizes occlusion, a green circle with a partial of red sector indicates incomplete occlusion (in this version of the software, the location of the red marks did not correspond with the exact spatial location of the leak), and a complete red circle symbolizes no occlusion. This described initial step of the occlusion tool is called the “baseline algorithm”. This step gives the physician feedback during appositioning of the balloon without the use of contrast-dye, angiography, or fluoroscopy. Once the baseline algorithm has confirmed a correct occlusion of the PV, a standard selective angiography was performed using a 1:1 mixture of saline (0.9%) with contrast medium (Imeron® with 300 mg/ml Iodine, Bracco, Germany) and cine acquisition (15 frames per second). This 3D-WBDIS utilizes a dye confirmation algorithm to monitor and assess in real-time the contrast injection. This step is potentially more accurate and sensitive to smaller residual PV to LA leaks. During, and for 12 s after the injection, a dissipation waveform graph which represents the average change in local dielectrics induced by the injected dye, as sensed by the SMC electrodes, is being displayed. The dissipation waveform graph, together with real-time coloration of the electrodes that have sensed the dye presence, give the physician further insight into the occlusion testing dynamics. If the graph forms a plateau after the injection, an optimal occlusion with stasis in the PV is demonstrated, whereas if the graph returns to baseline the occlusion is merely partial. Using the same interface the 3D-WBDIS gives a color-coded qualitative indication of the occlusion state, complete (green) or incomplete (red). If the positioning of the balloon is technically more difficult, e.g., due to the orientation of the PV in relation to the transseptal puncture, more than one occlusion attempt was performed. Deflation of the balloon or at least retraction was necessary as remaining contrast dye influences the accuracy of the occlusion tool. See Figure 1 for details of both algorithms of the occlusion tool.

ICE helped to determine if an antral balloon position was achieved. Color doppler imaging was used in case of doubtful occlusion on the discretion of the physician. The freeze was started if the PV was completely occluded based on the 3D-WBDIS and angiography (± ICE). See Figure 1 and the Supplementary Video 1 for details.

### Electro-Anatomical Mapping

EAM was performed using the 3D-WBDIS. The system was used for advancing multipolar catheters from the groin access to the RA, to visualize atrial anatomy and activation, to use the occlusion tool, for the analysis of LA substrate. Anatomy maps from the groin and the right atrium were acquired by a decapolar steerable diagnostic mapping catheter (Biosense Webster Inc., California, United States) or an irrigated RF tip ablation catheter (Thermocool® SF, non-navigational, unidirectional, Biosense Webster Inc., California, United States). The anatomy of the LA was obtained automatically by the system with the use of the circular 8-pole, 20 mm SMC. The initial LA/PV map was created in a straightforward, intermittent fashion, and immediately prior to and during balloon positioning at each PV ostium in a clockwise rotation (LSPV, LIPV, RIPV, RSPV). After complete CB PVI, a second LA/PV map was obtained continuously including activation and voltage information from the PV, PV ostia and the LA. Automated Local Activation Time (LAT) maps were acquired with the circular 20 mm SMC or point-by-point using the RF-tip catheter. The reference for the LAT was chosen according to the arrhythmia e.g., the CS catheter electrodes 5/6. LAT and propagation maps were used for the understanding of AT or atrial flutter. The auto-LAT settings were adjusted as necessary, e.g., CL stability ±20%, LAT stability at 5 ms. Catheter stability and distance between each LAT was activated. LAT values were only reevaluated in case of noise or premature beats (spontaneous or mechanically induced).

The R wave of the QRS complex was used as a reference for bipolar voltages (maximum annotation). Voltage mapping for the analysis of LA substrate was performed after CBA in sinus rhythm using the following definition: dense scar (<0.1 mV), moderate scar (0.1–0.5 mV), and healthy tissue (>0.5 mV) (22–24). In case of sinus bradycardia (heart rate <55 per minute), the map was acquired during atrial pacing with a cycle length (CL) of 600 ms from the proximal or mid CS with the same settings for voltage map acquisition as previously described. In those patients with additional AA which qualified for detailed mapping, voltage information was also used with those settings. Voltage information in AF was not used to tailor the ablation strategy.

Atrial premature stimulations were performed from the proximal and distal CS catheters with single, double, and triple extra-stimuli, and high-rate stimulation. If no sustained AA was induced, continuous intravenous isoproterenol (up to 0.24 mg/h) was used to identify extra-PV trigger and to enhance arrhythmia induction during programmed atrial stimulations following PVI.

### Radiofrequency Ablation of Additional Arrhythmias

The map after CBA was acquired using the SMC introduced through the CB and the 15F cryosheath (FlexCath Advance™, Medtronic Inc, USA) also in those patients with left ATs or atrial flutter. To prevent air embolisms with the use of a large sheath and a standard catheter, the cryosheath was exchanged over a stiff wire placed in the LSPV to a standard 8.5F steerable sheath (Agilis™ NxT steerable, Abbott, Illinois, United States). Additional ablation beyond PVI were performed using a standard unidirectional, non-navigated, D-curved, 3.5 mm, irrigated surround-flow radiofrequency (RF) mapping- and ablation catheter (Thermocool® SF, Biosense Webster Inc., California, United States). Irrigation flow rate was set at 2 (base rate), 8 (up to 30 watts) or 15 ml (>30 watts) per minute. RFA was performed with 20–35 watts. The duration of the application was left at the physician's discretion and ranged between 20 and 120 seconds.

The aim of RFA was to terminate the ongoing tachycardia and, depending on the underlying mechanism, to check for non-inducibility, completeness of lines (bidirectional block, pacing along the line, bipolar voltage and activation map following ablation) as required following the standards of invasive electrophysiology (1, 19). In this study, we neither apply the ablation of complex atrial electrograms in AF nor a LA substrate ablation strategy in sinus rhythm. If an ablation line from two electrically isolated obstacles seems necessary to terminate the arrhythmia, the design line of it would be created including nearby areas with atrial fractionations (broad signal with multiple deflections) and low-voltage (<0.5 mV).

### Post-procedure Care

Transthoracic echocardiography was performed in all patients to rule out pericardial effusion. Protamine was administered immediately before sheath removal in the lab. All patients were transferred to the normal ward which provides telemetric observation of the patients (continuous ECG, interval blood pressure measurement, pulsoximetry) for a minimum of 12 h following the procedure. Bedrest was arranged for 12 h.

Anticoagulation was continued for ≥3 months, and thereafter based on the individual CHA_2_DS_2_-VASc (Congestive heart failure, Hypertension, Age ≥75 years (doubled), Diabetes mellitus, prior Stroke or TIA or thromboembolism (doubled), Vascular disease, Age 65–74 years, Female sex category) score. All patients were empirically treated with proton-pump inhibitors for 4 weeks.

Scheduled follow-up was performed at 1, 3, and 6 months following the procedure and included symptom assessment, 12-lead ECG and Holter-studies.

### Follow-Up

Follow-up was performed in the outpatient clinic and/or in collaboration with the referring physician. During the ongoing Severe Acute Respiratory Syndrome Coronavirus type 2 (SARS-CoV2) pandemic, follow-up was additionally carried out by medical doctors via phone and/or videotelephony.

Freedom from AA defined as documented AF, atrial flutter (typical or atypical), AV-nodal-reentry-tachycardia, AV-reentry tachycardia or AT was defined as the efficacy endpoint of follow-up. If class I or III antiarrhythmic drugs were initiated or dose-increased, or pharmacological or electrical cardioversion was performed following ablation, this was also counted as a failure. For this short-term outcome analysis, outcome is presented with and without a 90-days blanking period.

### Statistical Analysis

Data were prospectively collected in the local database (Filemaker Pro®, Claris International Inc., California, United States). Data were analyzed with Microsoft Excel and SPSS 21 (both IBM, Armonk, New York, United States). Descriptive statistics were used. Categorical variables are expressed as numbers and percentages and continuous variables as means with standard deviations or as medians with interquartile range (IQR) following tests of normal distribution. Kaplan-Meier Survival Curves were calculated with SPSS software.

## Results

### Study Population

From March to June 2021, a total of 17 patients, mean age 68.8 ± 12.2 years, with complex AA defined above were prospectively included. Among those, five females were enrolled (29.5%). Determined by echocardiography, the LA, measured from anterior to posterior, was 45.5 ± 8.3 mm and the median ejection fraction was 60% (IQR 5). Persistent AF was documented in 52.9% of patients, and the median history of AF was 33 months with the median maximum duration of a single episode of 17 days (IQR 133). The median CHA_2_DS_2_-VASc Score was 3 (IQR 2). One patient demonstrated a left common ostium, three patients a right accessory PV, and two patients with previous LA procedures were included (1 RF PVI, 1 CB first generation 23 mm size). The baseline parameters are depicted in Table 1.

**Table 1:** Baseline characteristics.

**Baseline characteristics**	**Total**
*N* patients, %	17 (100)
Age, years	68.8 ± 12.2
Females, %	5 (29.5)
LA diameter^*^, mm	45.5 ± 8.3
Ejection fraction^*^, %	60 [5]
AF history, months	33 [79]
Persistent AF, %	9 (52.9)
EHRA symptom score	3 [1]
Prior pacemaker implantation, %	2 (11.8)
AAD I/III prior to the procedure, %	2 (11.8)
Number of electrical cardioversions	1 [2]
Number of episodes per year	20 [170]
Max. duration of a single AF episode, days	17 [133]
Hypertension, %	15 (88.2)
Hypertensive heart disease^*^, %	9 of 15 (60.0)
Mitral regurgitation ≥ grade 2, %	3 (17.7)
CAD, %	6 (35.3)
One-vessel CAD	2/6 (33.3)
Two-vessel CAD	1/6 (16.7)
Three-vessel CAD	0/6 (0.0)
Coronary sclerosis without stenosis	3/6 (50.0)
Prior myocardial infarction, %	0/6 (0.0)
Cardiomyopathy, %	1 (5.9)
Diabetes mellitus, %	1 (5.9)
Prior cerebrovascular event, %	1 (5.9)
Prior Aflut ablation, %	3 (17.7)
Hypothyroidism, %	2 (11.8)
Obstructive sleep apnea, %	5 (29.4)
Chronic kidney disease, %	2 (11.8)
GFR, ml/min	39.5 ± 12.0
CHA_2_DS_2_-VASc Score	3 [2]
BMI, kg/m^2^	27.40 [6.26]
Overweight (BMI > 25), %	7 (41.2)
Obesity (BMI > 30), %	1 (5.7)
Obesity grade 2 or 3 (BMI > 35), %	3 (17.7)
Common ostium, %	1 (5.7)
Accessory veins, %	3 (17.7)
Reablation	1 following RF-PVI
	1 following CBG1 23 mm

*N (%): number of patients and percentage; Mean ± Standard Deviation or Median with interquartile range [in squared brackets] as appropriate according to the test of normal distribution*.

*AAD, antiarrhythmic drug; AF, atrial fibrillation; BMI, body mass index; CAD, coronary artery disease; CBG, cryoballoon generation; CHA_2_DS_2_-VASc, Congestive heart failure, Hypertension, Age ≥75 years (doubled), Diabetes mellitus, prior Stroke or TIA or thromboembolism (doubled), Vascular disease, Age 65–74 years, Female sex category; EHRA, European heart rhythm association; LA, left atrium; GFR, glomerular filtration rate*.

* *Measured by transthoracic echocardiography*.

### Procedural Data

Procedural data are shown in Table 2. The left atrial procedure times were longer in those patients with additional tachycardia and ablation beyond PVI. Ablation results for CBA show a high rate of single shot isolation (85.7%), and 100% of acute PVI. The mean number of cryoapplication cycles and the total cryotime are depicted. Fluoroscopy times and dose area products were higher in patients with additional arrhythmias.

**Table 2:** Procedural characteristics per patient.

**Procedural results**	**Total**
*N* patients, %	17 (100)
Complex AF, %	100%
Persistent AF, %	9 (52.9)
Paroxysmal AF, %	8 (47.1)
Pts. with additional arrhythmias, %	5 (29.4)
+Right atrial flutter, %	2 (11.7)
+AVNRT, %	1 (5.7)
+Left atrial appendage tachycardia, %	1 (5.7)
+Left atrial flutter, %	1 (11.7) (two different mechanisms)
Total procedure time, min	135.2 ± 43.4
Total LA time, min	88 [40]
Total fluoroscopy time, min	20.3 ± 10.4
Total dose area product, cGy × cm^2^	1,100 [1,252]
Major complications, %	0 (0)
Minor complications, %	1 (5.7)
Type of complication	Tongue bite
**Cryo data**	
28 mm CB, %	17 (100)
Total Veins, %	70 (100)
Acute PVI, %	70 (100)
Number of TTI, %	50 (71.4)
Mean TTI, sec	38.5 (25)
Cryo procedure time, min	120 [43]
Cryo LA time, min	85 [30]
Cryo fluoroscopy time, min	17.77 ± 7.90
Cryo dose area product, cGy x cm^2^	1,002 [1,146]
Median esophageal temperature, °C	19.26 [7.87] *n* = 11
Number of angiographies	12 ± 5.53
**Occlusion tool and mapping data**	
Number of occlusion tool used, %	129 (100)
Occlusion tool used per PV	1.84 ± 0.96
Occlusion tool used per pts	7.59 ± 2.56
Occlusion tool successful, %	106 (82.2)
Angiography successful, %	109 (84.5)
Accuracy compared to angiography, %	119 (92.2)
Median number of maps per patient	3 [2]
Total maps	68
Median number of mapping points	251.0 (298.0)
Median map volume (milliliter)	52.8 (83.9)
**Freeze data**	
Number of freezes per patient	7 [3.5]
Number of freezes per PV	2 [1]
Single-shot success, %	60 (85.7)
Minimal balloon temperature, °C	−47 [8]
Ineffective freeze attempts, %	10 (7.8)
Freeze duration, sec	180 [0]
Intraprocedural reconnection, %	1 (1.4)
**RF data**	
RF procedure time, min	60 [60] *n* = 5
RF LA time, min	28 [76] *n* = 5
RF fluoroscopy time, min	8.60 ± 6.61 *n* = 5
RF dose area product, cGy × cm^2^	406 [467] *n* = 5
Number of RF applications	9.4 ± 7.54 *n* = 5
Maximal wattage, W	35 [45] *n* = 5

*AF, atrial fibrillation; Aflut, atrial flutter; AVNRT, atrioventricular nodal reentrant tachycardia; AT, atrial tachycardia; TTI, Time to Isolation; CB, cryoballoon; CBG, cryoballoon generation; LA, left atrium; PVI, pulmonary vein isolation; RF, radiofrequency; °C, degree Celsius; cGy, centigray; cm, centimeter*.

### Electro-Anatomical Mapping

**Figure 2:**
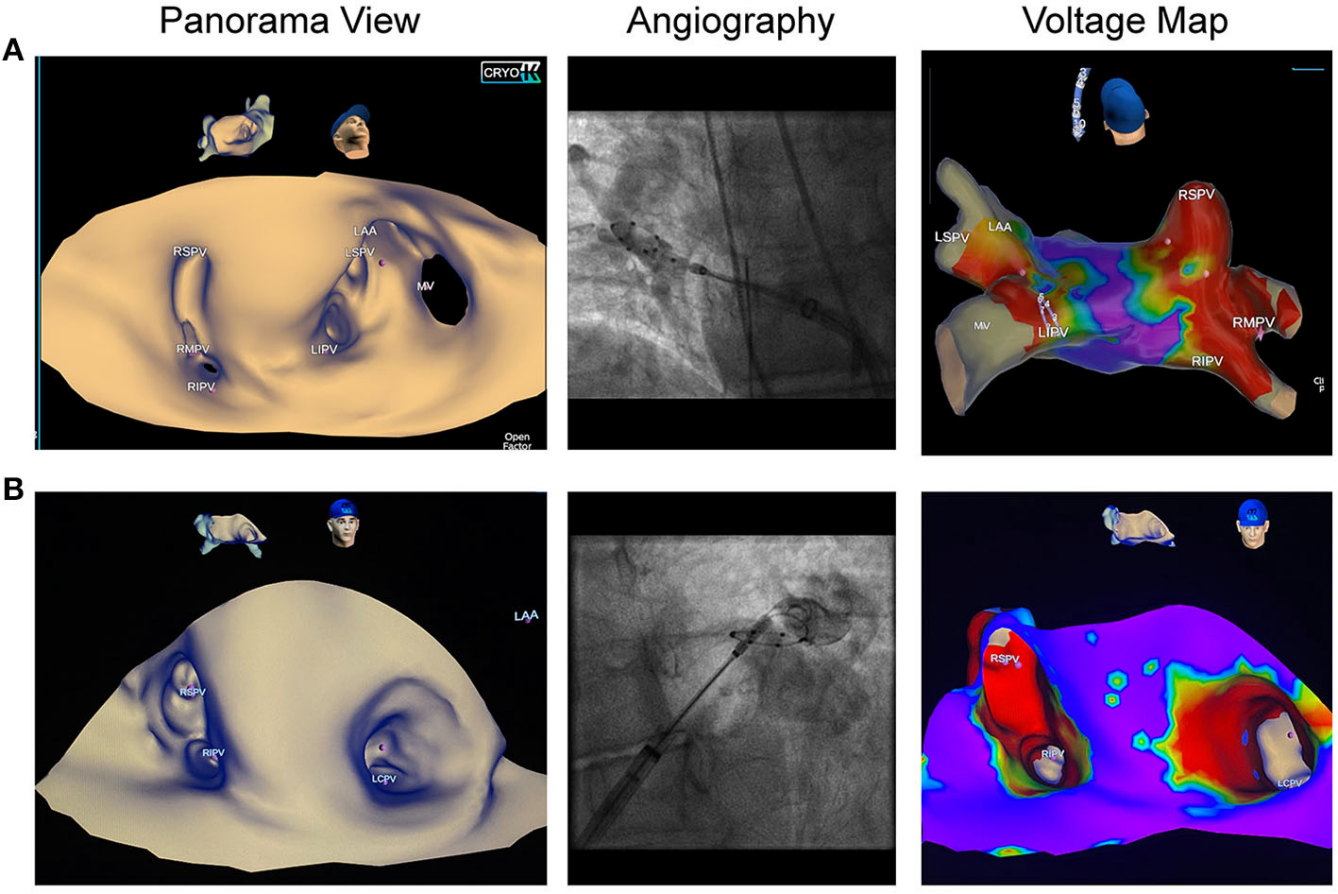
Pulmonary vein variants visualized by dielectric imaging and voltage mapping. Left atrial three-dimensional anatomy was acquired in sinus rhythm prior to cryoballoon ablation (CBA). Left sided pictures show the panoramic view, pictures in the middle show pulmonary vein (PV) angiographies, right sided images demonstrate voltage maps after CBA. **(A)** Patient referred for repeat AF ablation following radiofrequency PV isolation. Left atrial anatomy map shows a right accessory pulmonary vein, which was isolated by CBA. **(B)** Patient with de novo persistent atrial fibrillation shows a left common trunk with an ostial diameter of 26 mm. Voltage map was acquired after en-bloc cryoballoon isolation of the left common ostium and depicts wide antral isolation.

EAM with the SMC and 3D-WBDIS was feasible in all patients, and detailed anatomy was displayed prior to ablation. In four patients, PV variants were diagnosed (Figure 2, Table 1). Anatomical variants were confirmed by PV angiography and ICE. No pre-procedural evaluation of the PV anatomy was performed as a reference. Activation and voltage maps were achieved in all patients. We observed no specific difference between the mapping catheters. Anatomy mapping and activation mapping was faster with multipolar mapping catheters like the 8-pole 20 mm SMC and slower with the ablation catheter. No map shift was observed during any of the procedures.

During the CB applications, the SMC is placed near to the PV ostium and is commonly frozen. No dielectric information, and no electrograms are recorded after finalization of the occlusion tool algorithm, from the beginning to the completion of each freeze. Details on the purpose of each map, mapping results and benefits of the acquired maps are included in Supplementary Table 1.

### Evaluation of the Occlusion Tool

CBA of every PV (*n* = 70) was guided by the additional use of the occlusion tool. A total of 129 positioning maneuvers were evaluated (1.84/PV). The occlusion tool was used 31 (24.0%), 25 (19.4%), 33 (25.6%), 34 (26.4%), 2 (1.6%), 4 (3.1%) times in LSPV, LIPV, RIPV, RSPV, left common and accessory PV, respectively. The sensitivity of the occlusion tool defined as a “truly occluded PV” referenced to PV angiography (standard of care) with a complete occlusion (subjective grade 4 out of 4) as described before (26), was in total 94.5%, and 96.7, 90.0, 91.3, 96.7% for LSPV, LIPV, RIPV and RSPV, respectively. The specificity defined as a “truly non-occluded PV” as compared to angiography (grade of occlusion ≤ 3 out of 4) was in total 85%, and 100, 60, 90, 100% for LSPV, LIPV, RIPV, and RSPV, respectively. The positive predictive value (PPV) was in total 97.2% (true positive/true positive + false positive), and 100, 90, 96, 100% for LSPV, LIPV, RIPV, and RSPV, respectively. The negative predictive value (NPV) was in total 73.9% (true negative/true negative + false negative), and 50, 60, 82, 80% for LSPV, LIPV, RIPV, and RSPV, respectively.

### Ablation Beyond PVI

**Figure 3:**
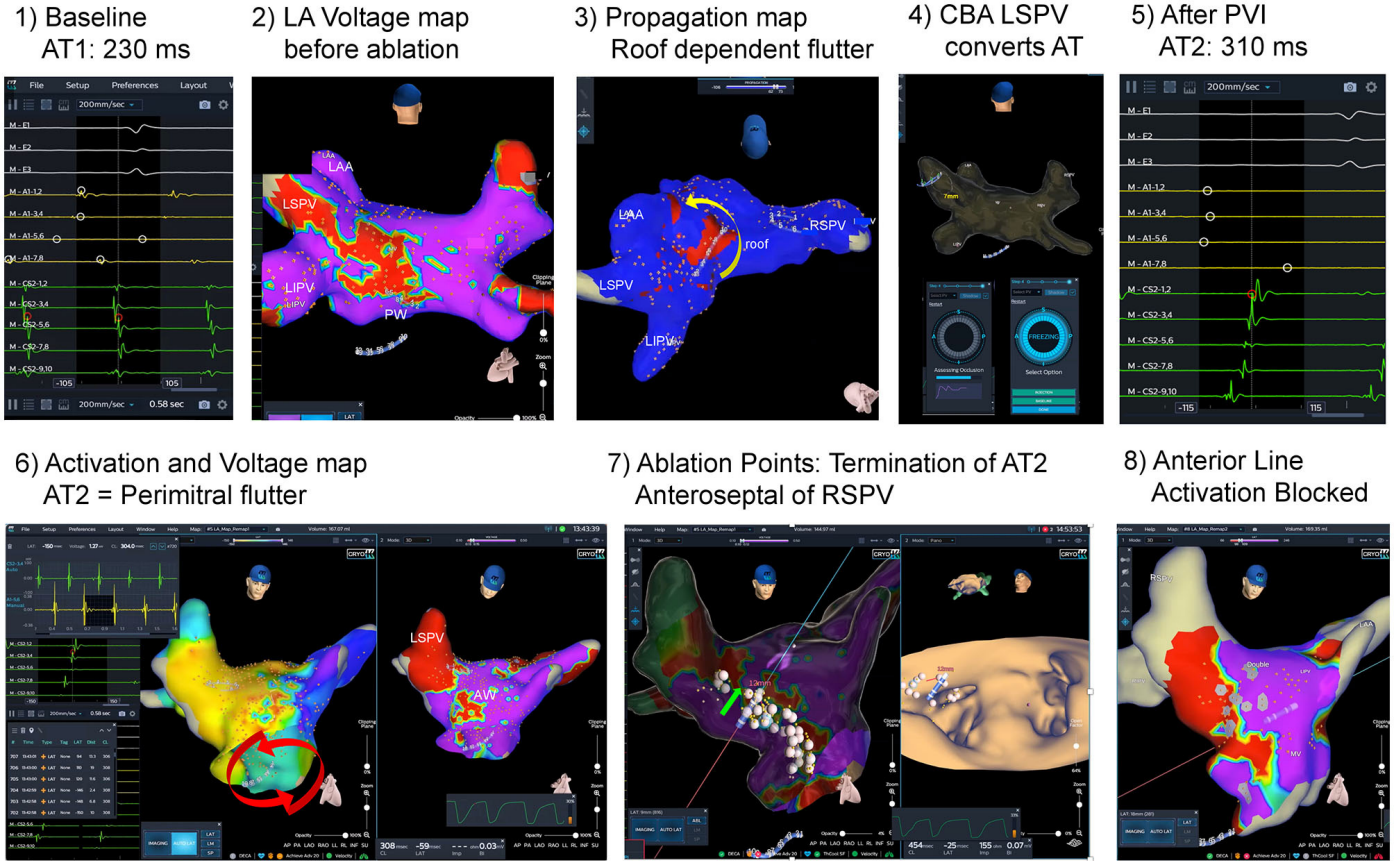
Atrial fibrillation and left atrial flutter ablation guided by dielectric imaging. Initial catheter ablation procedure guided by dielectric imaging. A 70-year old male patient with persistent atrial fibrillation (AF) and atypical atrial flutter. At baseline, left atrial (LA) flutter [atrial tachycardia 1(AT1): AT cycle length (CL) 230 ms] was documented (1). After transseptal puncture, acquired LA voltage map (2) shows low amplitudes and fractionation at the LA roof and posterior wall, and propagation map (see Image 3 and Supplementary Video 2) revealed roof dependent LA flutter around the lateral pulmonary veins (PV) from posterior to anterior wall (yellow arrow). Cryoballoon based isolation of the left superior PV converted the tachycardia to a AT2 [AT CL 310 ms, see (5)]. After complete PV isolation AT2 was mapped (6): activation map (Supplementary Video 3) demonstrated counterclockwise perimitral flutter, voltage map shows zones of fractionation and low voltage anterior and anteroseptal from the mitral annulus up to the anterior aspect of the right superior PV (RSPV). Modified anterior line was applied by irrigated radiofrequency ablation and AT2 terminated after completion of the line anteroseptal of RSPV. After a waiting time (20 min) completeness of line was evaluated by pacing along the line, and remap studies (8) during pacing in the distal and proximal part of the coronary sinus (CS). No atrial arrhythmias (AA) were inducible at the end of the procedure.

Additional RFA beyond PVI was performed in 5 of 17 patients (29.4%), and 6 arrhythmias were treated successfully (see Supplementary Table 1 for details). Two patients showed typical flutter. Right atrial isthmus ablation was performed, and bidirectional block was demonstrated. One male patient suffered from additional macro-reentrant tachycardias based on left atrial substrate: Left sided roof dependent flutter and perimitral flutter were both successfully mapped and ablated (see Figure 3, Supplementary Videos 2, 3). Bipolar voltage map of the LA in this patient demonstrated atrial fractionations defined as multiple deflections with a broad atrial signal and low-voltage amplitudes (<0.5 mV) at the anterior wall.

**Figure 4:**
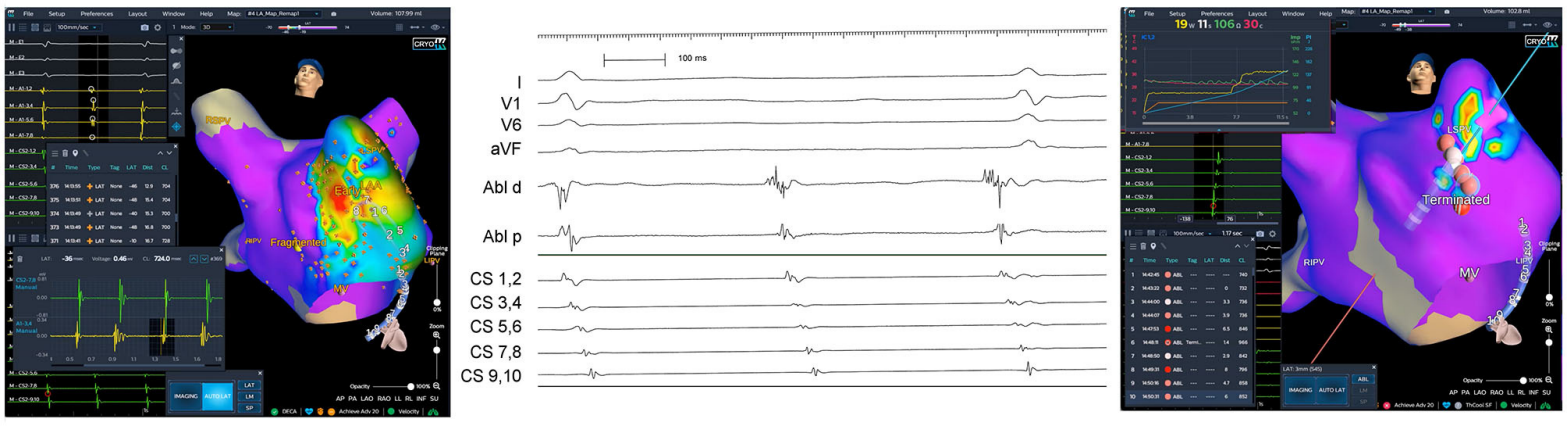
Focal atrial tachycardia from anterior aspect of the left atrial appendage. A female patient with recurrent paroxysmal atrial fibrillation (PAF) and atrial tachycardia (AT) was schedules for the initial electrophysiological study aiming at pulmonary vein isolation (PVI) to treat PAF and mapping of the AT in the same procedure. PVI was performed by imaging guided cryoballoon ablation as described before using the occlusion tool. Spontaneous left atrial focal tachycardia was observed under continuous isoproterenol challenge (0.12 mg/h). Activation map (left anterior oblique caudal view) showed the earliest activation (red) in the anterior aspect of the left atrial appendage (left). Activation pattern in the coronary sinus (CS) is from distal to proximal. Earliest atrial activation on the distal electrode of the ablation catheter (“Abl d,” middle part of the figure, electrogram tracing) was found at the anterior aspect of the left atrial appendage. First radiofrequency ablation terminated the ongoing AT and non-inducibility was achieved by three additional applications depicted in the right part of the figure.

One female patient suffered from additional triggered focal AT that originated from the left atrial appendage. Precise mapping and ablation were guided by the 3D-WBDIS (Figure 4). In one patient atrioventricular nodal reentry tachycardia was induced by premature stimulation. The arrhythmia was mapped and ablated using 3D-WBDIS.

One patient underwent CBA with first-generation 23 mm balloon in 2011 and was scheduled for repeat ablation because of arrhythmia recurrence. Circumscribed ostial PV isolation was demonstrated by 3D-WBDIS. No relevant substrate was determined, and no additional tachycardia was inducible. Therefore, big balloon reablation was the strategy. WACA was achieved by 28 mm fourth-generation CBA and demonstrated thereafter.

### Safety

No major complication occurred. One patient suffered from tongue bite while he was cardioverted in analogosedation before PVI. Minor amount of blood was sucked off successfully and the procedure was continued. Tongue bite was treated conservatively.

### Short-Term Outcome

**Figure 5:**
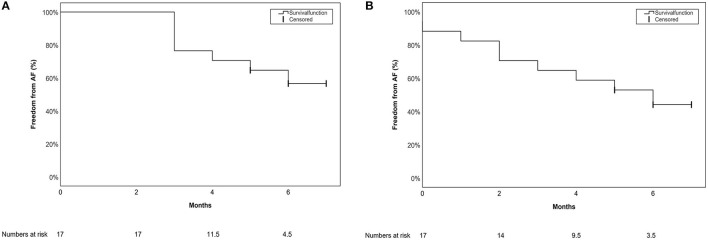
Single procedure short-term outcome for the primary endpoint depicted in a Kaplan-Meier Survival curve **(A)** with a 90-day blanking period **(B)** without a blanking period.

Freedom from AA after 6 (IQR 1) months of follow-up including a 90-day blanking period was documented in 10/17 (59%) patients, and 8/17 (47%) patients if no blanking period was applied. Freedom from AA was defined as documented AF, atrial flutter (typical or atypical), atrionodal-reentry-tachycardia, AV-reentry tachycardia or AT. In those cases with typical AF symptoms, highly symptomatic patients were also counted as a failure even if no documentation was achieved during the short follow-up time. Kaplan-Meier curves with and without a blanking period are presented in Figures 5A,B, respectively.

At last follow-up one of seventeen patients (5.9%) was still on an unchanged dose of flecainide, and 94.1% of patients were off AAD. In total, two electrical cardioversions of AF were successfully performed, both during the blanking period. All recurrences were AF related. No AT, atrial flutter, atrioventricular (nodal) reentry tachycardia was observed after discharge. No complications during the clinical course following discharge were observed.

## Discussion

To the best of our knowledge this is the first study evaluating the benefit of a novel 3D-WBDIS for the use in complex AA using CBA plus additional RFA as deemed appropriate in the initial ablation procedure.

The first step of our strategy consisted of a straightforward CBA based PVI which was guided by using the occlusion tool software module provided by the new imaging system in addition to conventional PV angiography. We were able to confirm the results of Cauti et al. (27) and Rillig et al. (17). The sensitivity and specificity of the occlusion tool were high. Although, sensitivity and specificity of the occlusion tool were similar in our study as compared to Cauti et al. (94.5 and 85% vs. 91 and 71%), the NPV in our study was slightly lower in the present investigation (74 vs. 88.6%). In particular, the left sided PV showed a lower NPV as compared to the right sided PV in our study (LSPV 50%, LIPV 60% vs. RIPV 82% and RSPV 80%). Interestingly, the specificity of the occlusion tool analyzed for each PV shows a lower value for LIPV (60%) as compared to the remaining PVs (LSPV 100%, RIPV 90%, RSPV 100%). Cauti et al. has previously shown that there is a predicted variation in specificity for each PV (27). Our results confirm the hypothesis that a variation might exist for the accuracy of the occlusion tool in the present version when approaching some veins such as the LIPV. However, this result should be interpreted with caution because of the limited number of applications and a potential learning curve with the system. The fact that a complete washout of remaining contrast dye is essential in case of a second PV angiography in the same vein must be considered by every operator if the occlusion tool is used to determine the quality of the balloon occlusion. Moreover, it remains unknown whether the PV angiography and the physician who interprets the angiogram (considered gold standard) might have missed very small gaps which could have potentially been detected by the sensitive 3D-WBDIS occlusion testing method.

The current version of the occlusion tool is validated for the exclusive use of the Arctic Front Advance™ cryoablation system (Medtronic Inc., Minneapolis, United States). The 3D-WBDIS is not validated for the POLARx™ cryoablation system (Boston Scientific Inc., Massachusetts, United States). Although the 3D-WBDIS was used in addition to the established standards of CBA, the time in LA for CBA was reasonably low.

The current use of the occlusion tool includes steps for baseline acquisition, positioning guided by the system and the confirmation of the occlusion by contrast dye injection and angiography (see Supplementary Video 1, Figure 1). Despite the consequent use of the occlusion tool software in this study, we still performed a contrast-dye angiography of each PV before each freeze. Schillaci et al. showed that a reduction in radiation exposure and dye use during CB ablation in patients with AF is possible (28). Further studies are necessary to demonstrate if the angiography can be omitted by the use of the occlusion tool. However, in our study the focus was set on the combination of CBA and RFA. Because the acquisition of a full LA anatomy takes about 5–10 min and this is not a prerequisite to use the occlusion tool, we decided to perform an intermittent LA/PV mapping while positioning the balloon over the SMC at each PV ostium. Complete LA map was continuously acquired following PVI to demonstrate the WACA, to analyze the substrate and the activation in each patient individually.

The use of the imaging system for CBA showed additional advantages as PV variants were visualized immediately, the level of antral PVI was demonstrated, and the physician was able to get additional information on LA voltage and activation characteristics as well as LA volumes in the initial ablation procedure.

The second step of our strategy was to identify and treat additional AA in the same procedure including changing the energy modality from CB based ablation to irrigated RF tip-catheter ablation. It must be mentioned that standard catheters were used without additional magnetic sensors in the tip of the tool. This allows a versatile usage of the mapping system. Mechanisms of AT and atrial flutter were identified, underlying substrate was studied and tachycardias were treated successfully. In the present first-generation of the 3D-WBDIS processing unit and software version 1.4.8, detailed anatomy can be provided using the non-contact assessment of dielectric gradients globally and locally with the system. However, LAT and voltage maps were determined by contact acquisition as in other established mapping systems, and the density of points is currently limited. In this study, no map shifts defined as inaccuracy of the 3D model >/=2 mm were observed. This might be an indication that the dielectric-based intra-body localization method is resilient to physiological changes.

In this study, only two patients with previous AF ablation procedures were studied and successfully ablated. Due to the limited number of LAT mapping points with 3D-WBDIS, we would prefer to treat PVI non-responders, candidates for AF substrate ablations, with ultra-high-density mapping systems. However, future enhancements of the system including the use of multielectrode mapping catheters, and automated high-density mapping capacity are awaited.

Although no air embolism was observed in our study, there might be a potential small risk for air embolism if the large 15F cryosheath (FlexCath Advance™, Medtronic) must be changed over the wire to the 8.5F steerable Agilis sheath (Abbott Inc., USA) to introduce a conventional irrigated tip RF catheter to the LA following PVI for RFA of additional left AAs. However, this change of the sheaths is mandatory as the manufacturer has only confirmed the compatibility of Freezor MAX™ and Arctic Front Advance™ cryoablation catheters with the FlexCath Advance™ sheath (all Medtronic Inc., United States). The use of other catheters has not been fully evaluated; therefore, the manufacturer does not recommend their use with FlexCath Advance™ sheath.

The ablation was performed without a contact-force sensing catheter in our study. There is evidence for higher rates of long-term durable PVI if contact-force information are used in an ablation index for PVI (29, 30). Here, PVI is performed by means of CBA which provides broad and wide circumferential PVI in a fast, safe, and effective single shot procedure (31, 32). It is unclear if contact-force sensing catheters are beneficial for the treatment of AT or atrial flutter. A randomized trial with blinded vs. unblinded contact-force information for left anterior line formation did not show a benefit of contact-force guided ablation (33). As contact-force has a fundamental role in effective lesion generation and is integrated in ablation index guided applications (34), the integration of contact-information, tissue analysis, and lesion assessment are a subsequent next steps for the novel system, and might be realized by the analysis of changes in the gradients of electrical fields during ablation in the future.

After a short-term follow-up of 6 months, ten of seventeen patients (59%) with complex AA were in stable sinus rhythm after a single 3D-WBDIS guided ablation including CBA and optional RFA in case of inducible additional atrial arrhythmias beyond AF. No recurrence of the ablated additional tachycardia was observed.

Outcome of an initial catheter ablation procedure in patients with complex AA as defined in this study does not exist. However, the published results in persistent AF should serve as a first reference to place our results into perspective. A recently published meta-analysis of RFA in persistent or long-standing persistent AF which included 113 studies and 18,657 patients demonstrated that the efficacy of a single AF ablation procedure is 43%. Moreover, they showed that the efficacy increased to 69% with the use of multiple procedures and/or anti-arrhythmic drugs (25). The prospective randomized STAR-AF II Trial did not show that additional ablation beyond PVI results in improved outcome in persistent AF (35). Although, additional arrhythmias targeted in this studies (focal AT from LAA, AVNRT, LA flutter) are often symptomatic, recurrent in its nature and might serve as extra-PV triggers for AF, it remains speculative, if patients benefit from the additional RFA treatment because the trial design did no provide a group for comparison. Evidence from repeat ablation following CBA and guided by ultra-high-density mapping revealed that PVI is still durable in up to 70% of symptomatic patients and PVI non-responders are older, mainly suffering from complex AA (8). And even after the second procedure these patients with complex AA demonstrate recurrences in 51% of patients after 12 months.

The use of 3D-WBDIS processing unit in combination with CBA requires the disposable body surface sensors and support by the clinical field specialist to use the innovative novel system. In those patients with inducible additional arrhythmias which were treated by means of RFA, an additional RF upgrade key was used to get access to the mapping and RFA tools of the system. An additional RF catheter became necessary only in those patients demonstrating sustained arrhythmias as a target for RFA following CBA. The choice of the RF ablation catheter (including resterilized catheters) was on the discretion of the physician as most of the commercially available catheters were already pre-qualified for this open platform system, and no special magnetic tracking or chipping is necessary for the use of the catheter in combination with the 3D-WBDIS. If RFA in LA was performed following CBA, an additional steerable sheath became necessary.

Three physicians were involved in the ablation procedures. Based on the initial experience with the present first-generation of the 3D-WBDIS processing unit (Software 1.4.8) and the evaluated strategy to treat complex AA, the following potential benefits of the strategy were identified:

1) Both, CB PVI and RFA of additional tachycardia were successfully guided by the 3D-imaging system.
2) Detailed anatomy visualization was achieved without direct tissue contact.
3) The use of the occlusion tool was feasible and will potentially reduce radiation in the future.
4) Voltage maps shows level of PVI following CBA and provide additional information of the LA substrate in the initial procedure.
5) LAT maps were useful for the physician to guide additional ablations beyond PVI.
6) The open platform allows the usage of any mapping/ablation catheter, and no special magnetic sensor containing catheters or chipped catheters were necessary.
7) No map shifts occurred which might indicate the resilience of the novel intra-body localization method.

The 3D-WBDIS has recently emerged, and potential fields of improvements have been identified by the users of the current version:

1) The number of mapping points is lower as compared to novel ultra-high-density mapping systems. The validation of high-density catheters e.g., mini-basket is recommended.
2) The option to change mapping catheters during the acquisition of a single map should be available, for example for those situations where additional points should be annotated immediately before RFA in a map which was acquired by the SMC before.
3) At the moment, only limited measurement tools are available (only distances), the user would recommend to also add measurement of areas, for example for the quantification of low-voltage zones.
4) Presently, no contact-force information is available from the system except those from the RF generator (impedance).
5) To demonstrate biatrial tachycardia, the system should provide display biatrial LAT and propagation maps.
6) The occlusion tool might be optimized by faster assessment of the occlusion (higher processor capacity awaited in the next processing unit release).
7) There is still an injection necessary for determining the quality of occlusion although the baseline algorithm without the injection is already a useful tool for balloon positioning. The user wonders if the accuracy of the system can be enhanced to provide similar accuracy with the baseline algorithm as compared to the injection algorithm.
8) The necessity to deflate or remove the balloon from PV before a second injection represents an additional maneuver in CBA guided by 3D-WBDIS. However, this step seems to be necessary as long as contrast-dye is used for the evaluation of the occlusion as the mixture of contrast-dye, saline and blood might remain in the partially occluded PV. Dielectric gradients might be weaker in those situations and could influence the accuracy of the occlusion tool. This can be prevented only if the balloon is deflated, the system is flushed with pure saline before a second positioning attempt, or no contrast dye is use with the occlusion tool (currently not approved).
9) Visualization of the CB itself and the cryosheath would help with positioning of the ablation system without the use of fluoroscopy or ICE.

Based on the results of this pilot study and from the perspective of the users of the system, subsequent larger, prospective studies should demonstrate if dielectric imaging guided ablation with a combined use of CBA for PVI and RFA for additional ablation in the initial ablation has an impact on major long-term outcomes. As the effort in the initial ablation procedure is higher as compared to a PVI only strategy, more data is necessary to demonstrate if the need for rehospitalizations, and repeat ablations, can be diminished.

## Limitations

Due to the nature of this observational pilot study with a small number of cases, inherent risk for bias exists. Larger studies with longer follow-up information are necessary to evaluate the risks and benefits of this approach. In this manuscript, investigational features of the novel mapping systems have been discussed. They have been clearly marked throughout the manuscript.

## Conclusions

Dielectric imaging can guide CB PVI and additional RFA for the treatment of AT and atrial flutter in patients with complex AA. The occlusion tool software allows non-fluoroscopic assessment of balloon positioning at the PV ostia and has the potential to reduce radiation exposure for patients and staff. Detailed anatomy of cardiac chambers is assessed by the analysis of gradients in electrical fields with and without direct tissue-catheter contact. Voltage and activation mapping allows the distinct analysis and treatment of additional AA. The open platform is the key for the combined use of different catheters and energy sources in the same procedure. Short-term outcome is favorable, however larger studies with longer follow-up are necessary to evaluate the benefits of the approach.

## Data Availability Statement

Beside the institutional registry database, no further closed or public database was used in this study. The institutional database was kept on the server of the Heart-Center Munich-Bogenhausen, Munich, Germany. The authors confirm that the data supporting the findings of this study are available within the article and/or its Supplementary Materials. The consent for publication of raw data was not obtained and dataset could in theory pose a threat to confidentiality. The reason why the consent for publication of raw data was not obtained is that personal health information was the objective of the study. The prepared datasets generated during and/or analyzed during the current study are available from FS on reasonable request.

## Ethics Statement

The studies involving human participants were reviewed and approved by Ethic Committee of the Bavarian State Medical Association (reference number 7/11140). The patients/participants provided their written informed consent to participate in this study.

## Author Contributions

FS, UD, and EH made the concept of the study. Data acquisition and analysis were performed by FS, JP, MW, and LR. YS was responsible for the technical correctness of the manuscript as a novel technology was discussed. The interpretation of the data was carried out by FS, UD, EH, and JP. FS and JP have drafted the work. UD, LR, YS, MW, and EH have substantively revised the manuscript. JP, UD, LR, YS, MW, EH, and FS have approved the submitted version (and any substantially modified version that involves the author contributions to the study) and have agreed both to be personally accountable for the author's own contributions and to ensure that questions related to the accuracy or integrity of any part of the work, even ones in which the author was not personally involved, are appropriately investigated, resolved, and the resolution documented in the literature. All authors contributed to the article and approved the submitted version.

## Conflict of Interest

FS received honoraria for lectures from Philips GmbH, Medtronic GmbH, and Bristol-Myers-Squibb, outside the submitted work; educational support from Pfizer. UD reports honoraria for lectures from Medtronic Inc., outside the submitted work. EH is head of the department; the department received compensation for participation in clinical research trials outside the submitted work from Abbott, Bayer, Biotronik, Boehringer Ingelheim, Edwards, Elixier, Medtronic, and Stentys. YS is employed by EPD Solutions (a Philips Company). The remaining authors declare that the research was conducted in the absence of any commercial or financial relationships that could be construed as a potential conflict of interest.

## Publisher's Note

All claims expressed in this article are solely those of the authors and do not necessarily represent those of their affiliated organizations, or those of the publisher, the editors and the reviewers. Any product that may be evaluated in this article, or claim that may be made by its manufacturer, is not guaranteed or endorsed by the publisher.

## Supplementary Material

Supplementary Table 1 Mapping and ablation data. A, Anatomy; Acti, Activation; AFlut, atrial flutter; AT, atrial tachycardia; CG, catheter guiding; CBA, cryoballoon ablation; CTIA, cavotricuspid isthmus ablation; D, Demonstration of complete PVI; E, evaluation of complete PVI, OT, occlusion tool, Pt, patient; SP, slow pathway; V, tissue voltage information.

Supplementary Video 1 (Occlusion Tool) This video of the 3D-WBDIS system was created during the use of the occlusion tool in the left superior pulmonary vein (PV) (software version 1.4.8). The arrangement of the windows is on the discretion of the physician. In the upper left hand corner the occlusion tool window is placed. Next to it, the 3D anatomy model is shown (middle, upper row), and on the upper right hand corner the panoramic view of the left atrium is located. In the lower row the electrograms are displayed left sided. On the lower right hand corner either the corresponding intracardiac echocardiography live view or the PV angiography is shown. Both are not integrated into the 3D-WBDIS but were added to this video to give a reference for the quality of balloon occlusion.

Supplementary Video 2 (related to Figure 3, image 3) The video of the 3D-WBDIS screen shows the activation of the atrial tachycardia 1 (AT1) with a cycle length of 230 ms. The screen has three windows next to each other. Electrograms (left), activation map (0:00–0:27 min) or propagation map (0:27–1:03 min) (middle), voltage map (0:00–0:50 min) and panoramic view propagation map (0:50–1:03 min) (right). The orientation of the 3D models is show by an icon (head with hat). The video demonstrates roof dependent left atrial flutter around the lateral pulmonary veins (PV) from posterior to anterior wall (yellow arrow). The corresponding voltage map shows zones of voltages <0.5 mV at the roof close to the left superior vein).

Supplementary Video 3 (related to Figure 3, image 6) The video of the 3D-WBDIS screen shows the activation of the atrial tachycardia 2 (AT2) with a cycle length of 310 ms. The screen has three windows next to each other. Electrograms (left), activation map (0:00–0:23 min) or propagation map (0:23–1:00 min) (middle), voltage map (right). The orientation of the 3D models is show by an icon (head with hat). The video demonstrates perimitral left atrial flutter in a counterclockwise direction. The corresponding voltage map shows zones of voltages <0.5 mV anterior and anteroseptal of the left atrium).

## Abbreviations

3D: three dimensional
3D-WBDIS: Three Dimensional Wide-Band Dielectric Imaging System
AA: Atrial Arrhythmias
AF: Atrial Fibrillation
AT: Atrial Tachycardia
CB: Cryoballoon
CBA: Cryoballoon Ablation
CL: Cycle Length
CS: Coronary Sinus
ECG: Electrocardiography
EAM: Electro-anatomical Mapping
ICE: Intracardiac Echocardiography
IQR: Interquartile Range
LA: Left Atrium
LAT: Local Activation Time
LIPV: Left Inferior PV
LSPV: Left Superior PV
NPV: negative predictive value
PAF: paroxysmal AF
PPV: positive predictive value
PV: Pulmonary Vein
PVI: Pulmonary Vein Isolation
RIPV: Right Inferior PV
RF: radiofrequency
RFA: Radiofrequency Ablation
RSPV: Right Superior PV
SMC: Spiral Mapping Catheter
TTI: Time-To-Isolation
WACA: Wide Antral Catheter Ablation.
