# Supplementary material for: Catheter Ablation in Complex Atrial Arrhythmias: Pilot Study Evaluating a 3D Wide-Band Dielectric Imaging System
Source: Front Cardiovasc Med. 2022 Jan 28;8:817299. doi: 10.3389/fcvm.2021.817299 (PMC8831840; doi:10.3389/fcvm.2021.817299)
Supplement: Supplementary Table 1 — Mapping and ablation data. A, Anatomy; Acti, Activation; AFlut, atrial flutter; AT, atrial tachycardia; CG, catheter guiding; CBA, cryoballoon ablation; CTIA, cavotricuspid isthmus ablation; D, Demonstration of complete PVI; E, evaluation of complete PVI, OT, occlusion tool, Pt, patient; SP, slow pathway; V, tissue voltage information. [file Table_1.docx]

**Supplement Table 1: Mapping and ablation data**

| **Map #** | **Pt #** | **Pt Map #** | **Purpose** | **Chamber** | **Type of mapping** | **Condition** | **Cycle length for map acquision (ms)** | **Number of mapping points** | **Mapping volume (mL)** | **Benefit of map** | **Ablation** | **Success** |
| --- | --- | --- | --- | --- | --- | --- | --- | --- | --- | --- | --- | --- |
| 1 | 1 | 1 | Access Map | Access | A | n/a |  |  | 25.51 | A, CG | - | - |
| 2 | 1 | 2 | RA Map | RA | A, Acti, V | SR | 1100 | 161 | 104.79 | A, CG | - | - |
| 3 | 1 | 3 | CS Map | CS | A | SR |  |  | 5.27 | A, CG | - | - |
| 4 | 1 | 4 | LA Map baseline | LA | A, Acti, V | SR | 1100 | 436 | 108.84 | A, CG, V, Demonstration of RMPV, 4/5 w. reconnection  CBA, OT | PV-Reisolation | All PV isolated |
| 5 | 1 | 5 | LA Map post ablation | LA | A, Acti, V | SR | 812 | 497 | 108.84 | E |  | D |
| 6 | 2 | 1 | Access Map | Access | A | n/a |  |  | 9.91 | A, CG | - | - |
| 7 | 2 | 2 | RA Map | RA | A | SR |  |  | 24.48 | A, CG |  |  |
| 8 | 2 | 3 | For CBA | LA | A | SR |  |  |  | A, V, CG, CBA, OT | PVI | All PV isolated |
| 9 | 2 | 4 | After CBA | LA | A, Acti, V | SR | 1050 | 187 | 77.03 | E | - | D |
| 10 | 3 | 1 | Access Map | Access | A |  |  |  | 4.9 | A, CG | - | - |
| 11 | 3 | 2 | RA Isthmus | RA | A |  |  |  | 22.75 | A, CG, guiding RFA | CTIA | Bidirectional block |
| 12 | 3 | 3 | For CBA | LA | A, Acti, V | SR | 1150 | 239 | 27.94 | A A, V, CG, CBA, OT | PVI | All PV isolated |
| 13 | 3 | 4 | After CBA | LA | A, Acti, V | SR | 800 | 293 | 28.58 | E |  | D |
| 14 | 4 | 1 | Access Map | Access | A | SR |  |  | 4.07 | A, CG | - | - |
| 15 | 4 | 2 | RA Map | RA | A | SR |  |  | 19.88 | A, CG |  |  |
| 16 | 4 | 3 | For CBA | LA | A, Acti, V | SR | 1070 | 396 | 22.51 | A, V, CG, CBA, OT | PVI | All PV isolated |
| 17 | 4 | 4 | After CBA | LA | A, Acti, V | SR | 1070 | 371 | 36.71 | E | - | D |
| 18 | 5 | 1 | Access Map | Access | A | SR |  |  | 8.45 | A, CG | - | - |
| 19 | 5 | 2 | RA Map | RA | A | SR |  |  | 14.8 | A, CG |  |  |
| 20 | 5 | 3 | For CBA | LA | A, Acti, V | SR | 1070 | 119 | 38.39 | A, V, CG, CBA, OT | PVI | All PV isolated |
| 21 | 5 | 4 | After CBA | LA | A, Acti, V | SR | 1100 | 454 | 83.8 | E | - | D |
| 22 | 6 | 1 | Access Map | Access | A | SR |  |  | 46.35 | A, CG | - | - |
| 23 | 6 | 2 | RA Map | RA | A | SR |  |  | 42.5 | A, CG |  |  |
| 24 | 6 | 3 | For CBA | LA | A, Acti, V | SR | 940 | 187 | 78.67 | A, V, CG, CBA, OT | PVI | All PV isolated |
| 25 | 6 | 4 | After CBA | LA | A, Acti, V | SR | 700 | 356 | 133.27 | E | - | D |
| 26 | 7 | 1 | Access Map | Access | A | n/a |  |  |  | A, CG | - | - |
| 27 | 7 | 2 | RA Map | RA | A |  |  |  | 60.23 | A, CG | - | - |
| 28 | 7 | 3 | LAT Map For CBA | LA | A, Acti, V | AT1 | 230 | 1093 | 164.83 | A, V, CG, CBA, OT, Acti: roof dependent LA flutter | PVI | All PV isolated, LAT converted into 2. LAT |
| 29 | 7 | 4 | After CBA LAT Map | LA | A, Acti, V | AT2 | 310 | 789 | 138.14 | E, A, V, CG, Acti: perimitral LA flutter; guiding RFA | RFA: modified anterior line | Termination, non-inducibility |
| 30 | 7 | 5 | LAT | LA | A, Acti, V | Paced | 600 | 281 | 148.46 | E | - | Line of Block |
| 31 | 8 | 1 | Access Map | Access | A |  |  |  | 17.06 | A, CG |  |  |
| 32 | 8 | 2 | RA Map | RA | A, Acti, V | SR | 1000 | 63 | 60.06 | A, CG |  |  |
| 33 | 8 | 3 | For CBA | LA | A, Acti, V | SR | 1200 | 290 | 88.23 | A, V, CG, CBA, OT | PVI | All PV isolated |
| 34 | 8 | 4 | After CBA | LA | A, Acti, V | SR | 1200 | 251 | 136.05 | E | - | D |
| 35 | 9 | 1 | RA Map | RA | A | Paced | 850 |  | 24.57 | A, CG |  |  |
| 36 | 9 | 2 | For CBA | LA | A, Acti, V | Paced | 860 | 210 | 67.25 | A, V, CG, CBA, OT | PVI | All PV isolated |
| 37 | 9 | 3 | After CBA | LA | A, Acti, V | Paced | 860 | 454 | 129.91 | E | - | D |
| 38 | 10 | 1 | Access Map | Access | A |  |  |  | 32,59 | A, CG |  |  |
| 39 | 10 | 2 | RA Map | RA | A |  |  |  | 35.41 | A, CG |  |  |
| 40 | 10 | 3 | For CBA | LA | A, Acti, V | SR | 880 | 225 | 66.47 | A, V, CG, CBA, OT | PVI | All PV isolated |
| 41 | 10 | 4 | After CBA | LA | A, Acti, V | SR | 880 | 568 | 141.93 | E | - | D |
| 42 | 11 | 1 | Access Map | Access | A |  |  |  | 16.11 | A, CG |  |  |
| 43 | 11 | 2 | CS Map | CS | A |  |  |  | 2.71 | A, CG |  |  |
| 44 | 11 | 3 | For CBA | LA | A, Acti, V | AT | 360 | 307 | 113.49 | A, Acti, V, CG, CBA, OT | PVI | All PV isolated |
| 45 | 11 | 4 | After CBA | LA | A, Acti, V | AT | 360 | 526 | 120.11 | E, A, Acti, V, CG, guiding RFA | LAA Tachycardia | Successful ablation anterior aspect of LAA |
| 46 | 12 | 1 | RA Map | RA | A |  |  |  | 32.85 | A, CG |  |  |
| 47 |  | 2 | For CBA | LA | A, Acti, V | SR | 1050 | 93 | 98.18 | A, V, CG, CBA, OT | PVI | All PV isolated |
| 48 |  | 3 | After CBA | LA | A, Acti, V | SR | 1050 | 540 | 214.4 | E | - | D |
| 49 | 13 | 1 | Access Map | Access | A |  |  |  | 8.45 | A, CG |  |  |
| 50 | 13 | 2 | RA Map | RA | A |  |  |  | 8.78 | A, CG |  |  |
| 51 | 13 | 3 | For CBA | LA | A, Acti, V | SR | 1100 | 48 | 44.34 | A, V, CG, CBA, OT | PVI | All PV isolated |
| 52 | 13 | 4 | After CBA | LA | A, Acti, V | SR | 1100 | 93 | 99.61 | E | - | D |
| 53 | 14 | 1 | Access Map | Access | A |  |  |  | 10.2 | A, CG |  |  |
| 54 | 14 | 2 | RA Map | RA | A |  |  |  | 34.65 | A, CG |  |  |
| 55 | 14 | 3 | For CBA | LA | A, Acti, V | Paced | 600 | 1515 | 184.16 | A, V, CG, CBA, OT | PVI | All PV isolated |
| 56 | 14 | 4 | After CBA | LA | A, Acti, V | Paced | 600 | 1155 | 218.27 | E | - | D |
| 57 | 15 | 1 | RA Map | RA | A |  |  |  | 45.01 | A, CG |  |  |
| 58 | 15 | 2 | For CBA | LA | A, Acti, V | SR | 930 | 189 | 119.81 | A, V, CG, CBA, OT | PVI | All PV isolated |
| 59 | 15 | 3 | After CBA | LA | A, Acti, V | SR | 920 | 210 | 196.79 | E | - | D |
| 60 | 16 | 1 | RA Map | RA | A |  |  |  | 75.19 | A, CG |  |  |
| 61 | 16 | 2 | For CBA | LA | A, Acti, V | Paced | 500 | 427 | 59.57 | A, V, CG, CBA, OT | PVI | All PV isolated |
| 62 | 16 | 3 | After CBA | LA | A, Acti, V | Paced | 500 | 133 | 91.18 | E | - | D |
| 63 | 16 | 4 | RA Map for RFA | RA | A, Acti, V | Induced AVNRT | 310 | 53 | 23.74 | A, CG, guiding RFA | SP ablation | Elimination of SP |
| 64 | 17 | 1 | RA Map | RA | A |  |  |  | 22.82 | A, CG |  |  |
| 65 | 17 | 2 | For CBA | LA | A, Acti, V | SR | 1100 | 57 | 95.11 | A, V, CG, CBA, OT | PVI | All PV isolated |
| 66 | 17 | 3 | After CBA | LA | A, Acti, V | SR | 1100 | 169 | 117.36 | E | - | D |
| 67 | 17 | 4 | RA Map typical AFlut | RA | A, Acti, V | RA AFlut | 260 | 111 | 53.35 | A, Acti, V, CG, guiding RFA | CTIA | Bidirectional block |
| 68 | 17 | 5 | RA Map after CTIA | RA | A, Acti, V | Paced | 600 | 117 | 52.24 | E |  | Demonstration of bidirectional block |
|  |  |  |  |  |  |  | **900.0 (477.5)** | **251.0 (298.0)** | **52.8 (83.9)** |  |  |  |

A: Anatomy; Acti: Activation; AFlut: atrial flutter; AT: atrial tachycardia; CG: catheter guiding; CBA: cryoballoon ablation; CTIA: cavotricuspid isthmus ablation; D: Demonstration of complete PVI; E: evaluation of complete PVI, OT: occlusion tool, Pt: patient; SP: slow pathway; V: tissue voltage information
